# Testosterone treatment prevents most gonadectomy-induced metabolic and skeletal deficiencies in female mice

**DOI:** 10.3389/fendo.2026.1940100

**Published:** 2026-10-16

**Authors:** Robin E. Kruger, Hannah Yohannes, Evelyn Cho, Lainie Eisner, Daniel R. Pfau, Ruth K. Chan-Sui, Nicole Migotsky, Andre Monteiro da Rocha, Megan L. Killian, Molly B. Moravek, Vasantha Padmanabhan, Ariella Shikanov

**Affiliations:** 1 Department of Obstetrics and Gynecology, University of Michigan, Ann Arbor, MI, United States; 2 Department of Pediatrics, University of Michigan, Ann Arbor, MI, United States; 3 Department of Biomedical Engineering, University of Michigan, Ann Arbor, MI, United States; 4 Department of Orthopaedic Surgery, University of Michigan, Ann Arbor, MI, United States; 5 Department of Internal Medicine, Division of Cardiovascular Medicine, University of Michigan, Ann Arbor, MI, United States; 6 Department of Molecular & Integrative Physiology, University of Michigan, Ann Arbor, MI, United States; 7 Reproductive Medicine Associates of Michigan, Livonia, MI, United States

**Keywords:** metabolism, ovariectomy, sex hormones, skeletal, testosterone

## Abstract

Ovarian hormone depletion, whether due to ovariectomy, primary ovarian insufficiency, menopause, or gonadotoxic therapies, is associated with metabolic and skeletal dysfunction, while hyperandrogenic states such as polyendocrine metabolic ovarian syndrome are also linked to increased cardiometabolic risk. However, whether testosterone exacerbates, mitigates, or modifies the consequences of ovarian hormone loss remains poorly understood. This study examined whether testosterone treatment ameliorates ovariectomy-induced metabolic and skeletal impairments using an adult female mouse model. Eight-week-old CD-1 mice underwent sham surgery or ovariectomy and were implanted subcutaneously with capsules containing 10 mg testosterone or empty control capsules, yielding four experimental groups: Blank+Intact (n=7), Blank+OVX (n=7), Testosterone+Intact (n=7), and Testosterone+OVX (n=8). Each treatment combination resulted in hormonal milieus distinct to each group. After six weeks of treatment, testosterone mitigated ovariectomy-induced hyperglycemia and impaired glucose tolerance but did not prevent dyslipidemia or adipocyte hypertrophy (two-way ANOVA with Tukey’s *post hoc* test, p<0.05). Testosterone treatment also increased circulating triglycerides, heart weight, femoral trabecular bone density, and radial strength independent of ovarian status. These findings indicate that testosterone compensates for some, but not all, metabolic and skeletal consequences of ovarian hormone loss, revealing distinct roles for androgens and estrogens in metabolic homeostasis. This may inform clinical management of ovarian insufficiency, menopause, hyperandrogenic disorders, and testosterone-based therapies.

## 1 Introduction

Sex steroid hormones are central regulators of metabolic, cardiovascular, and skeletal health. In males and females, estrogens and androgens coordinate energy balance, lipid metabolism, glucose regulation, and bone remodeling through actions in key metabolic tissues, including adipose, liver, skeletal muscle, and bone (1–4). Estradiol deficiency, whether caused by ovariectomy, primary ovarian insufficiency, menopause, or gonadotoxic therapies, is associated with increased adiposity, dyslipidemia, insulin resistance, cardiovascular disease, and osteoporosis (5–8). While the consequences of estradiol loss are well-documented, the impacts of testosterone on female cardiometabolic health and its interaction with ovarian factors remain less understood.

Androgens play important regulatory roles in metabolism and bone physiology. In males, testosterone contributes to the regulation of glucose utilization especially in skeletal muscle, as well as insulin signaling, adiposity, lipolysis and lipogenesis (9). In females, the metabolic effects of testosterone are less clearly defined despite an established connection in pathogenic contexts. Hyperandrogenic conditions such as polyendocrine metabolic ovary syndrome (PMOS, formerly PCOS) are strongly associated with adverse metabolic outcomes including increased risk of obesity, diabetes mellitus, and cardiovascular disease (10). Although the etiology of metabolic pathologies in PMOS is not well understood and outcomes are heavily influenced by environmental factors, it raises the question of whether elevated testosterone alone may exacerbate metabolic disease. Conversely, testosterone also has anabolic effects on bone and skeletal muscle, which may confer cardiometabolic and musculoskeletal benefits (11, 12). Importantly, testosterone can also be aromatized to estradiol, further complicating efforts to distinguish androgen-specific effects from those mediated indirectly through estrogen signaling.

It remains unclear how chronic testosterone exposure may influence metabolism and skeletal health in individuals with ovaries and how testosterone may compensate for ovarian hormones, particularly in the context of diminished or absent ovarian function. Clinical studies of exogenous testosterone administration in patients show inconsistent effects on lipid profiles, insulin sensitivity, body weight, and blood pressure (13–17). Interpretation of these studies is often constrained by retrospective or cross-sectional designs and confounding environmental or physiological variables including heterogeneity in hormone regimens, participant age, treatment duration, baseline metabolic status, and endogenous gonadal function. Furthermore, none of these studies have examined potential differences in outcomes between patients with and without endogenous ovaries. Controlled animal models therefore provide a valuable translational approach for isolating the direct physiological effects of testosterone and ovarian hormone loss on metabolism and skeletal health. We addressed this question using a validated mouse model of chronic testosterone administration that reproduces endocrine features associated with elevated androgens (18).

The objective of this study was to determine whether chronic testosterone exposure influences metabolic and skeletal outcomes and if these effects are modulated by the presence of ovaries using a mouse model. We hypothesized that retaining endogenous gonads would protect against adverse metabolic and skeletal outcomes even during exogenous testosterone exposure, while testosterone treatment would prevent some deleterious consequences of ovariectomy. By comparing testosterone-treated and untreated mice with or without ovaries, we aimed to define the relative contributions of androgen signaling and ovarian-derived factors to metabolic regulation and skeletal homeostasis. We found that testosterone treatment was sufficient to prevent ovariectomy-induced hyperglycemia and reduced glucose tolerance. Testosterone exposure also increased femoral trabecular bone density and radial strength independent of ovary presence, supporting anabolic effects of androgen signaling on the skeleton. However, ovariectomy remained associated with elevated cholesterol levels and increased adipocyte size despite testosterone treatment, indicating that ovarian factors continue to play a protective role in lipid metabolism. Together, these findings demonstrate that testosterone can compensate for some, but not all, physiological consequences of ovarian hormone loss and highlight distinct roles for androgens and ovarian-derived hormones in regulating metabolic and skeletal homeostasis.

## 2 Methods

### 2.1 Mouse husbandry

Adult female CD-1 mice (Envigo) were co-housed five in a cage under 12-hour light/dark cycle conditions and given food and water *ad libitum* unless otherwise indicated. Mice were left undisturbed for one week after arrival prior to experimental manipulations. All animal procedures were approved by the Institutional Animal Care & Use Committee at the University of Michigan (PRO00012925).

### 2.2 Mouse surgery and terminal measures

One week before surgery, daily vaginal cytology samples were collected for one week to confirm estrous cycling as described (18). At eight weeks old, mice were given a pre-surgery analgesia (5 mg/kg carprofen, subcutaneous injection), anesthetized (2-4% isoflurane, inhaled) and implanted subcutaneously with a silastic capsule containing either 10 mg testosterone powder (“Testosterone”) or an empty control capsule (“Blank”). At the same time, mice underwent either dorsal ovariectomy (“OVX”) or a sham surgery (“Intact”). This produced four treatment groups (n=8/group): Blank+Intact, Blank+OVX, Testosterone+Intact, and Testosterone+OVX (**Figure 1A**). One mouse died from complications of surgery, and one died from natural causes during the treatment period. After six weeks of treatment, mice were fasted overnight for 12 hours before being weighed and euthanized in diestrus. Euthanasia was performed via decapitation under anesthesia (5% isoflurane, inhaled). One control mouse that was euthanized in estrus was excluded from further analyses. Trunk blood was collected for terminal serum measurements. The left inguinal fat pad, left perigonadal fat pad, uterus, liver, and whole heart were collected from each mouse and weighed. For liver and fat pads, the tissue was cut in half and one half flash-frozen in liquid nitrogen. The remaining halves of the fat pads were fixed overnight in 4% paraformaldehyde (Thermo Scientific, J61899.AK). Remaining liver tissue was fixed in Bouin’s fixative (Ricca Chemical 1120-16) overnight. Hearts were left intact, with half of the samples flash-frozen (n=3–4 per group) and the other half fixed in 4% paraformaldehyde (n=3–4 per group). For uterine tissue, one uterine horn was fixed overnight in Bouin’s fixative. Femurs and radii were also collected, with one femur fixed for 48 hours in 4% paraformaldehyde.

**Figure 1:**
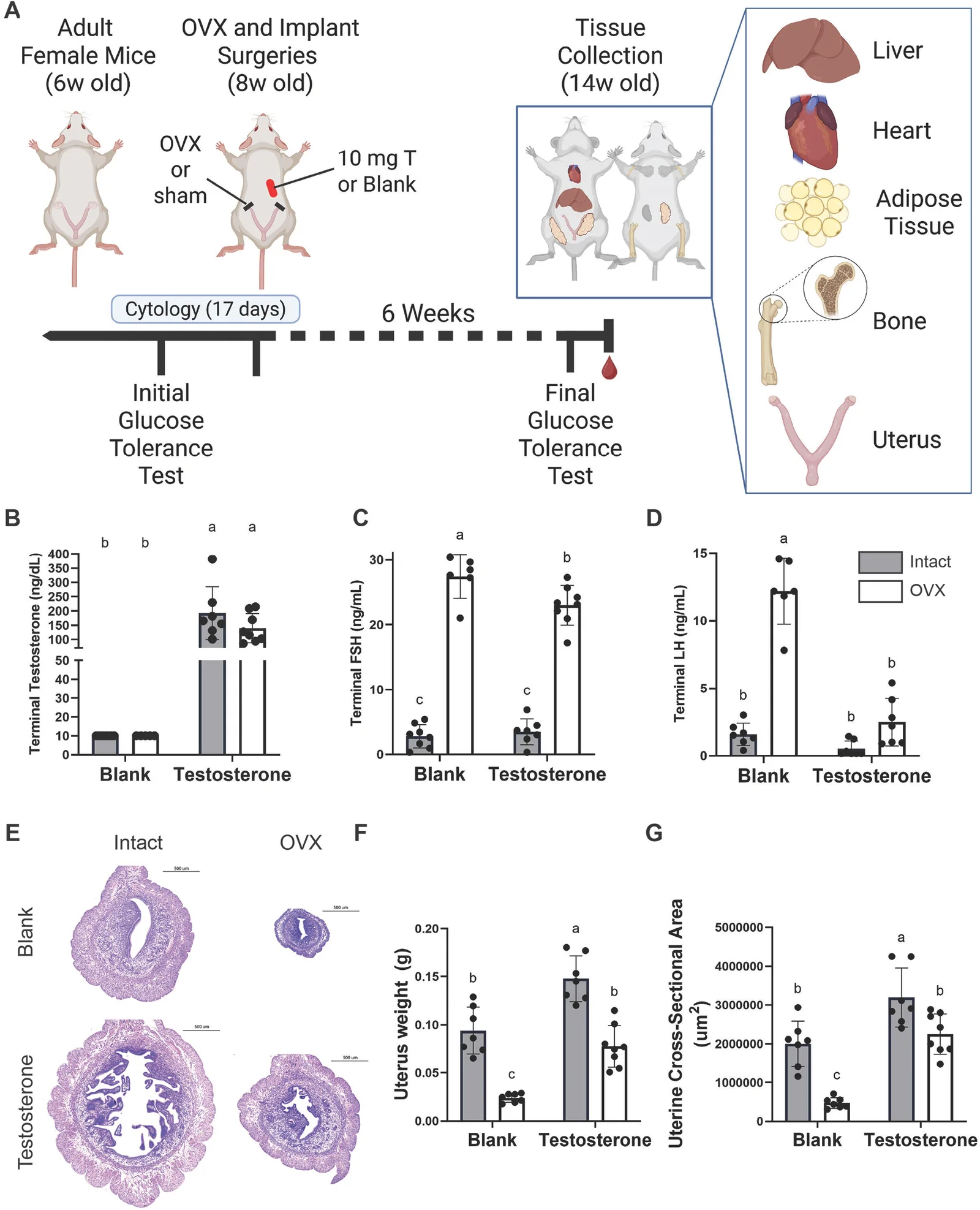
Experimental approach. **(A)** Timeline of experimental investigation. Eight-week-old CD-1 intact and ovariectomized mice received subcutaneous implants either containing 10 mg T or empty control capsules. After six weeks of treatment, metabolic, skeletal, and uterine tissues were collected for analysis. Terminal circulating levels of **(B)** testosterone, **(C)** FSH, and **(D)** LH. **(E)** Representative terminal uterine histological cross-sections stained with hematoxylin and eosin. **(F)** Terminal uterine weights. **(G)** Quantification of average uterine cross-sectional area from histological sections. Statistical differences between groups as assessed by two-way ANOVA are represented by distinct letters (p<0.05). Scale bars = 500 µm.

### 2.3 Glucose tolerance testing

Glucose tolerance testing was conducted at eight weeks old (before surgery) and 14 weeks old (after six weeks treatment). Mice were fasted overnight for 12 hours. After fasting, blood was collected from tail vein nicks and fasted glucose levels were measured using a FreeStyle Freedom Lite blood glucose monitor (Abbott). Mice were then injected intraperitoneally with 0.7 g/kg of 100 mg/mL D-glucose solution (Sigma). Glucose readings from tail vein blood were taken at intervals of 15, 30, 45, 60, 90, and 120 minutes after glucose injection.

### 2.4 Hormone and metabolic serum analysis

Terminal cardiac blood collected after six weeks of treatment was kept at 4 °C overnight, centrifuged for 10 min (8100g), and the collected serum stored at −20 °C. Serum testosterone, luteinizing hormone (LH), and follicle stimulating hormone (FSH) were measured by the Ligand Assay and Analysis Core Facility, University of Virginia Center for Research in Reproduction. FSH was measured by Ultra-Sensitive Mouse & Rat FSH ELISA (19), sensitivity range 0.016–8 ng/mL. LH was measured by Ultra-Sensitive Mouse & Rat LH ELISA (20), sensitivity range 0.016–4 ng/mL. Serum was diluted 1:20 before FSH measurement and 1:10 before LH measurement; reported values reflect undiluted hormone levels. Total testosterone was measured by IBL ELISA, sensitivity range 10 – 1, 600 ng/dL.

Metabolites were measured by the Michigan Diabetes Research Center Chemistry Laboratory at the University of Michigan (RRID: SCR_026768). Fasted glucose, triglycerides, HDL and total cholesterol were measured on a Randox Daytona Plus chemistry analyzer. Glucose: Randox GL8319, sensitivity range 0.35 – 61.9 mmol/L. Triglycerides: Randox TR8332, sensitivity range 12.4–1000 mg/dl. HDL-Cholesterol: Randox CH8311, sensitivity range 7.1–129 mg/dl. Total cholesterol: Randox CH8310, sensitivity range 23.2–618 mg/dl. Fasted insulin was measured by a double-antibody radioimmunoassay (RIA, modified from Linco Research, Inc.) using a 125I-Human insulin tracer (Millipore Sigma), a rat insulin standard (Novo), guinea pig anti-rat insulin first antibody (Millipore Sigma), and a goat anti-guinea pig gamma globulin (Antibodies Inc.)-PEG second antibody. Counts were measured using a Perkin Elmer Wizard 1470 gamma counter. Limit of sensitivity for the assay is 1.5 µU/ml.

### 2.5 Histology

After overnight fixation in 4% paraformaldehyde (Thermo Scientific, J61899.AK), all tissues were washed in 50% ethanol, then stored in 70% ethanol at 4 °C until taken for further analysis. Samples were processed, paraffin-embedded, and sectioned at Histology Core in the Microscopy & Image Analysis Laboratory at the University of Michigan. Adipose tissue was sectioned at 5 μm with three sections per slide and one in every four slides was collected for analysis for a total of 10 slides per sample. Uterine and heart tissue were serially sectioned at 5 μm with three sections per slide. One in every three slides was stained with hematoxylin (Abcam, AB220365) and eosin (Ricca Chemical, 1120-16), as in Pfau et al. (28). Slides were mounted with Permount resin-based mounting medium (Fisher, SP15-500), then imaged on a Leica DM 1000 LED microscope at 5X magnification (uterus) or 20X magnification (adipose and liver). For all histological analyses, four sections per sample were analyzed and reported values are averages of the four measurements per sample. Analyzed sections were at least 40 μm apart for uterus and heart tissue and at least 170 μm apart for adipose and liver tissue. Adipocyte size in adipose tissue was measured by the ImageJ Adiposoft package version 1.16 (21). The software automatically identified adipocytes which were manually edited and verified before final analysis. The cross-sectional area of the uterus was measured in FIJI version 2.16.0 (ImageJ, 22) by manual selection of the entire cross-section. Heart tissue was sectioned longitudinally to procure cross-sectional images containing all four chambers of the heart. Heart sections were whole-slide imaged with a high-resolution scanner (Leica Aperio AT2) at 20X magnification. The cross-sectional area of the ventricular chambers was measured in FIJI by manual selection of the entire ventricular cross-section.

### 2.6 Picro-sirius red staining

Hepatic fibrosis was assessed in fixed liver sections by Picro-sirius Red (PSR) staining. Tissue sections of 5 µm thickness were deparaffinized in two xylene washes for five minutes each and rehydrated through a graded ethanol series (100% twice, 95% twice, and 80% once; five minutes each). Four sections at least 170 µm apart were stained with picro-sirius red solution (Sigma-Aldrich 43665) for one hour at room temperature, followed by two washes in acidified water (1% glacial acetic acid, Sigma-Aldrich 64-19-7) for five minutes each. Slides were subsequently dehydrated in three washes of 100% ethanol and two washes of xylenes for five minutes each. Finally, sections were mounted using a resin-based mounting medium (Permount).

### 2.7 Oil Red O staining

To evaluate hepatic lipid accumulation, Oil Red O staining was performed on cryopreserved liver tissue. Immediately after euthanasia, liver samples were flash-frozen in liquid nitrogen and stored at −80 °C. Tissues were frozen in an embedding medium consisting of 10% sucrose (Sigma-Aldrich S1888) and 90% optimal cutting temperature (OCT) compound (Fisher 23730571). Prior to sectioning, embedded samples were equilibrated at −20 °C for 30–60 minutes and cryosectioned at a thickness of 10 µm. Nine sections at least 20 µm apart were rehydrated as needed with water, mounted on slides, and air-dried. Sections were fixed in ice-cold 4% paraformaldehyde for 30 minutes, followed by three washes in distilled water for five minutes each. Samples were then incubated in 60% isopropanol (Fisher BP2618) for five minutes and stained with Oil Red O solution (Sigma-Aldrich O0625) for 20 minutes at room temperature. After staining, sections were rinsed in 60% isopropanol for five minutes and washed four times with distilled water. Slides were counterstained with five dips in hematoxylin, mounted using an aqueous mounting medium, Fluoromount-G (Southern Biotech 0100-01), and sealed with clear nail polish (Ted Pella 114-7).

### 2.8 Wheat-germ agglutinin staining

Slides containing fixed cardiac tissue were prepared for staining with Trilogy (Sigma 920P) according to the manufacturer’s protocol. Briefly, four sections at least 40 µm apart were incubated in Trilogy in an electric pressure cooker for 15 minutes on “HIGH” setting. Samples were then rinsed for two minutes in hot Trilogy and washed three times in PBS at room temperature. The samples were stained with wheat lectin conjugated to FITC (Sigma L4895) at 1:50 dilution for one hour in the dark. After staining, samples were washed six times in PBS (10 min each) and mounted with aqueous mounting media (Fluoromount G). Fluorescently-labeled samples were imaged on a Leica SP8 confocal microscope using an HC PL APO CS2 20x/0.75 IMM objective with 2X digital zoom and 4X line averaging. Presented images are maximum intensity projections of 8-10 µm z-stack with 1 µm step size. Cardiomyocyte size was quantified using HeartJ package in FIJI (23). Tolerance was defined as 12 and ROIs defining individual cardiomyocytes were manually edited for completeness before final analysis.

### 2.9 Nano-computed tomography

Right femurs were dissected, fixed in 4% paraformaldehyde for 48 hours at 4 °C, and stored in 70% ethanol. Femurs were scanned using a Phoenix Nanotom M system (GE; 8.0 μm voxel; 80 kV; 400 μA; 0.381 mm aluminum filter; average parameter of 3; integration time of 500 ms) with hydroxyapatite calibration (1.69 mg/cc; Gammex). Scans were reconstructed using Datos|x software (GE) and uploaded into Dragonfly3D software (Object Research Systems) for processing. The cortical volume of interest (VOI) was defined as 10% of the total bone length distal to the third trochanter, and the trabecular VOI was defined as 10% of the total bone length at the distal metaphysis. Cortical and trabecular properties were calculated from each VOI following established methods (24).

### 2.10 Bone biomechanics (three point bending)

Right radii were dissected and stored in saline-soaked gauze at -20 °C. Prior to mechanical testing, radii were embedded in 2% agar and imaged using a SKYSCAN micro-computed tomography system (Bruker). Cross-sectional geometry was identified at the radius midshaft in Dragonfly3D and used to calculate the moment of inertia and maximum distance from the neutral axis to the periosteal surface. On the day of mechanical testing, samples were measured with calipers and centered onto supports with a span length of 7 mm attached to a uniaxial mechanical testing frame (Biomomentum). Radii underwent 10 cycles of sinusoidal preconditioning to 0.05 mm displacement at 0.1 Hz followed by 60 s of rest at 0 mm displacement. Radii were then loaded to failure at 0.05 mm/s. Bending stiffness and maximum load were calculated for each bone in R, and elastic modulus and maximum stress were calculated using engineering beam theory (25).

### 2.11 Statistical analyses

All statistical analyses were performed in GraphPad Prism 10.4.1 for Windows (GraphPad Software) by a researcher blinded to the treatments. Normality for all analyses was determined using a Shapiro-Wilk test. Significance was determined by two-way ANOVA with testosterone and gonad presence as factors, followed by Tukey’s *post hoc* test adjusted for multiple comparisons (n = 7-8/group; alpha = 0.05). Outliers detected by ROUT with Q = 1% were excluded from analysis. Frequency analysis for adipocyte size was conducted with a frequency distribution using bin sizes of 200 μm to create a histogram fitted with a Gaussian best fit curve. Pairwise comparisons in binned distributions were performed by pairwise Monte Carlo chi-square tests (B = 100, 000) with Holm correction for multiple comparisons (adjusted p<0.05). Chi-square tests were performed in RStudio (v. 2026.01.2 + 418).

## 3 Results

### 3.1 Effects of testosterone treatment on reproductive outcomes in ovary-intact and ovariectomized mice

Testosterone treatment increased circulating testosterone levels in both the ovary-intact and OVX groups to the range observed in male mice (**Figure 1B** (26, 27),). OVX groups exhibited elevated FSH compared to Intact groups as expected, but only Blank+OVX had elevated LH (**Figures 1C, D**). Blank+OVX mice showed expected uterine regression, in contrast to the Testosterone+OVX group which did not differ from controls in uterine area or weight. In Testosterone+Intact animals, uterine weight and area were increased as previously observed for this model (Pfau et al. (28), **Figures 1E–G**). All ovariectomized and T-treated mice ceased estrous cycling within three days of surgery (**Supplementary Figure 1**). These outcomes confirmed the efficacy of the treatments and validated the approaches for further investigation.

### 3.2 Testosterone treatment prevented OVX-induced glucose intolerance without affecting body weight

The effects of ovariectomy on obesity-related and general metabolic parameters were modified by testosterone treatment. While neither T-treatment nor OVX significantly impacted weight gain over the 6-week treatment period (**Figure 2A**; **Supplementary Figure 2A**), they had differential effects on glucose tolerance. Blank+OVX mice displayed impaired glucose tolerance and hyperglycemia without a proportional change in insulin that was not observed in the Testosterone+OVX group (**Figures 2B–E**). Relative to dyslipidemia, both Testosterone groups showed a trend (which achieved significance only in the Testosterone+OVX group) toward elevated blood triglyceride levels (**Figure 2F**). Finally, both OVX groups displayed higher total cholesterol and HDL levels than Intact groups (**Figures 2G, H**). As Testosterone+OVX mice exhibited normal glucose parameters but abnormal lipid profiles, these data suggest that testosterone treatment mitigates gonadectomy-induced glucose metabolism defects but not dyslipidemia.

**Figure 2:**
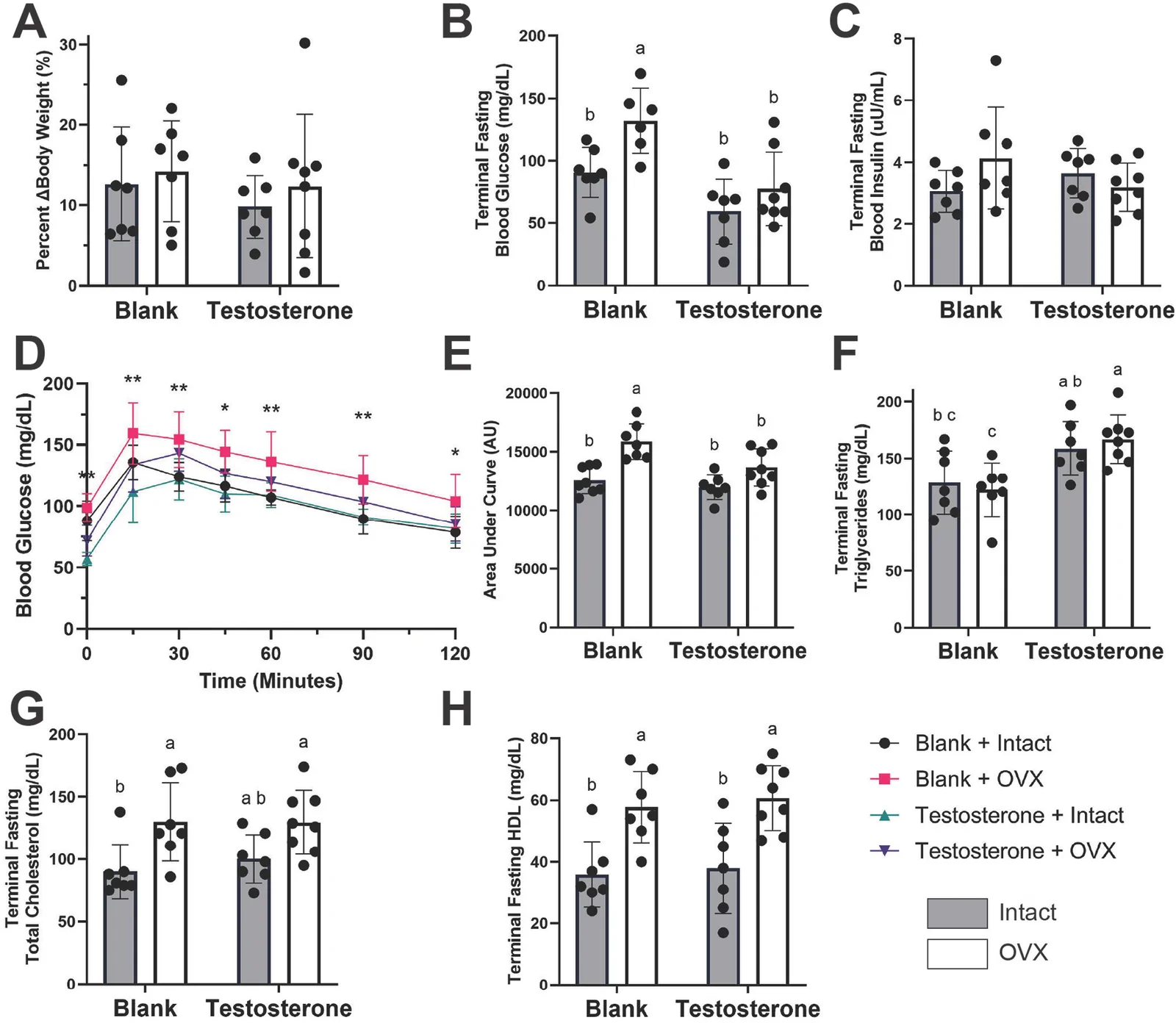
Effects of testosterone treatment on body weight and metabolic variables in ovary-intact and ovariectomized mice. **(A)** Percent change in total body weight over the 6-week treatment period. **(B)** Fasted terminal circulating glucose and **(C)** insulin levels. **(D)** Average glucose levels over time during glucose tolerance test after 6 weeks of treatment. Statistical differences were evaluated separately at each timepoint. **(E)** Area under the curve for glucose tolerance test results. **(F)** Fasted terminal levels of circulating triglycerides, **(G)** total cholesterol, and **(H)** high-density lipoprotein. Statistical differences between groups as assessed by two-way ANOVA are represented by asterisks or distinct letters (p<0.05). *p<0.05 **p<0.01.

### 3.3 Testosterone treatment improves fat redistribution in ovary-intact mice

Testosterone-mediated fat redistribution was dependent on gonad presence in both subcutaneous (inguinal) and visceral (perigonadal) depots. Analysis of terminal inguinal (**Figures 3A–D**) and perigonadal (**Figures 3E–H**) fat pads showed decreased fat pad weight and decreased median adipocyte size in Testosterone+Intact mice. However, this effect of testosterone on adipose tissue was not observed in the Testosterone+OVX group, suggesting that gonad retention is required for testosterone-mediated fat redistribution. Interestingly, OVX increased adipocyte size more in inguinal fat than perigonadal fat, though testosterone affected both adipose tissue types similarly (**Figures 3B–D, F–H**). As most collagen in adipose tissue remained membrane-associated across treatments, collagen quantification reflected the observed differences in surface area-to-volume ratio in adipocytes between groups but did not display evidence of widescale fibrosis (**Figures 3I–J, 3K–L**).

**Figure 3:**
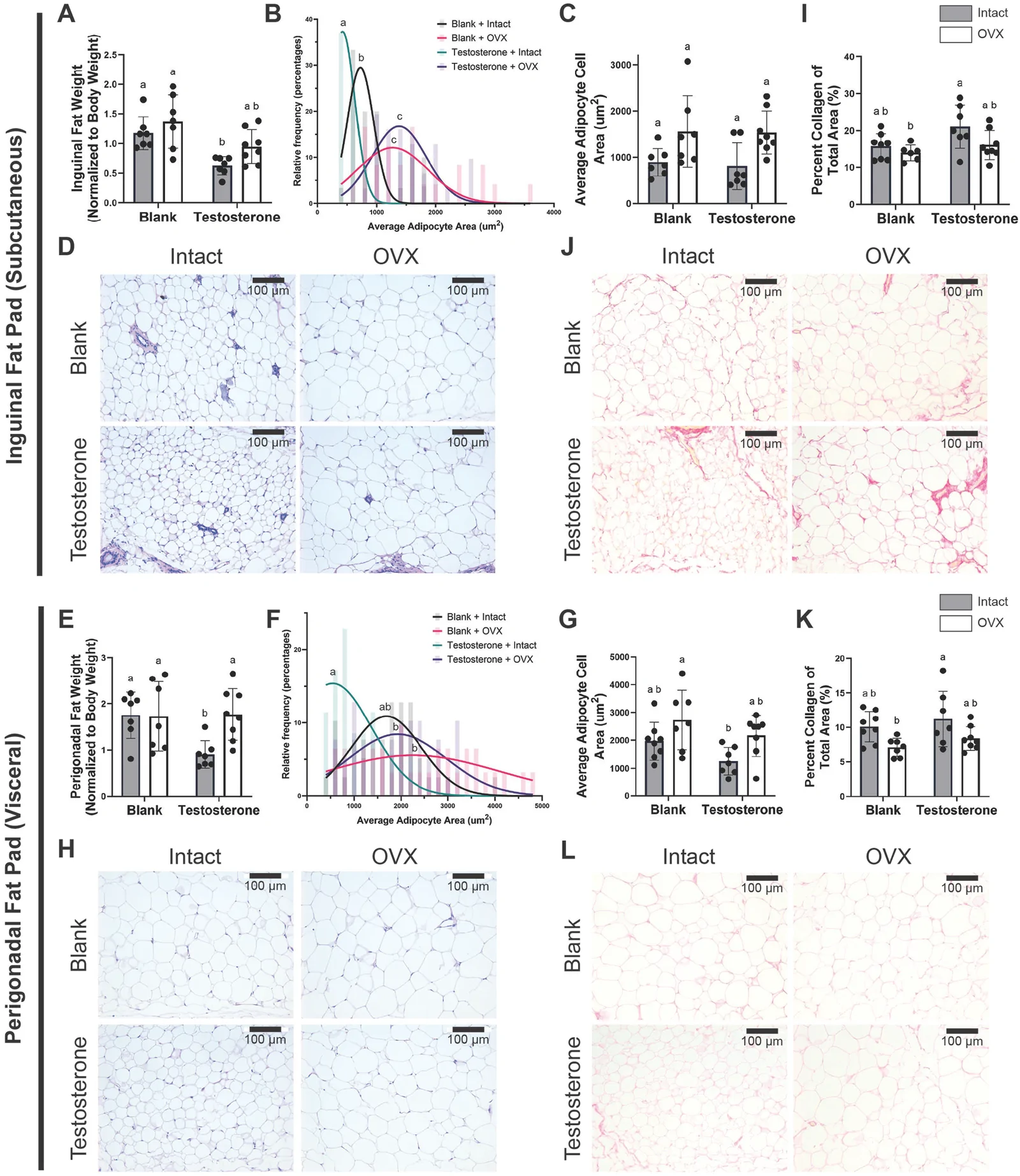
Effects of testosterone treatment on lipid homeostasis in ovary-intact and ovariectomized mice. **(A)** Terminal inguinal fat pad weights. **(B)** Histogram of frequency of adipocyte sizes binned in groups of 200 µm^2^, where peaks of Gaussian curves indicate median inguinal adipocyte size. **(C)** Average inguinal adipocyte area. **(D)** Representative H&E-stained sections of inguinal fat pads from each group. **(E)** Terminal perigonadal fat pad weights. **(F)** Histogram of frequency of adipocyte sizes binned in groups of 200 µm^2^, where peaks of Gaussian curves indicate median perigonadal adipocyte size. Pairwise comparisons in binned distributions were performed by pairwise Monte Carlo chi-square tests (B = 100, 000) with Holm correction for multiple comparisons (adjusted p<0.05). **(G)** Average perigonadal adipocyte area. **(H)** Representative H&E-stained sections of perigonadal fat pads from each group. **(I)** Percent collagen of analyzed area in inguinal fat. **(J)** Representative PSR stained slides of inguinal fat pads. K) Percent collagen of analyzed area in perigonadal fat. **(L)** Representative PSR stained slides of perigonadal fat pads. Statistical differences between groups as assessed by two-way ANOVA are represented by distinct letters (p<0.05). Scale bars represent 100 µm. (OVX, ovariectomy; H&E, hematoxylin and eosin; PSR, picrosirius red).

### 3.4 Testosterone treatment increases heart size independent of ovary presence

Testosterone treatment significantly increased heart weight in both ovary-intact and OVX mice compared to untreated controls (**Figures 4A, B**; **Supplementary Figures 2B, C**). This increase in heart weight was supported by congruent trends toward increased heart longitudinal area and left ventricular cardiomyocyte size, though statistical power was reduced for these comparisons as only half of the heart samples were available for histological analysis. (**Figure 4C–E**). Cardiomyocyte size was too variable in Testosterone+OVX mice to draw definitive conclusions regarding the interaction of ovary presence and testosterone treatment. Neither testosterone treatment nor OVX significantly impacted heart fibrosis (**Supplementary Figures 2D, E**). Similarly, in hepatic tissue, neither testosterone treatment nor OVX significantly impacted liver weight, collagen deposition, or lipid content (**Supplementary Figure 3**).

**Figure 4:**
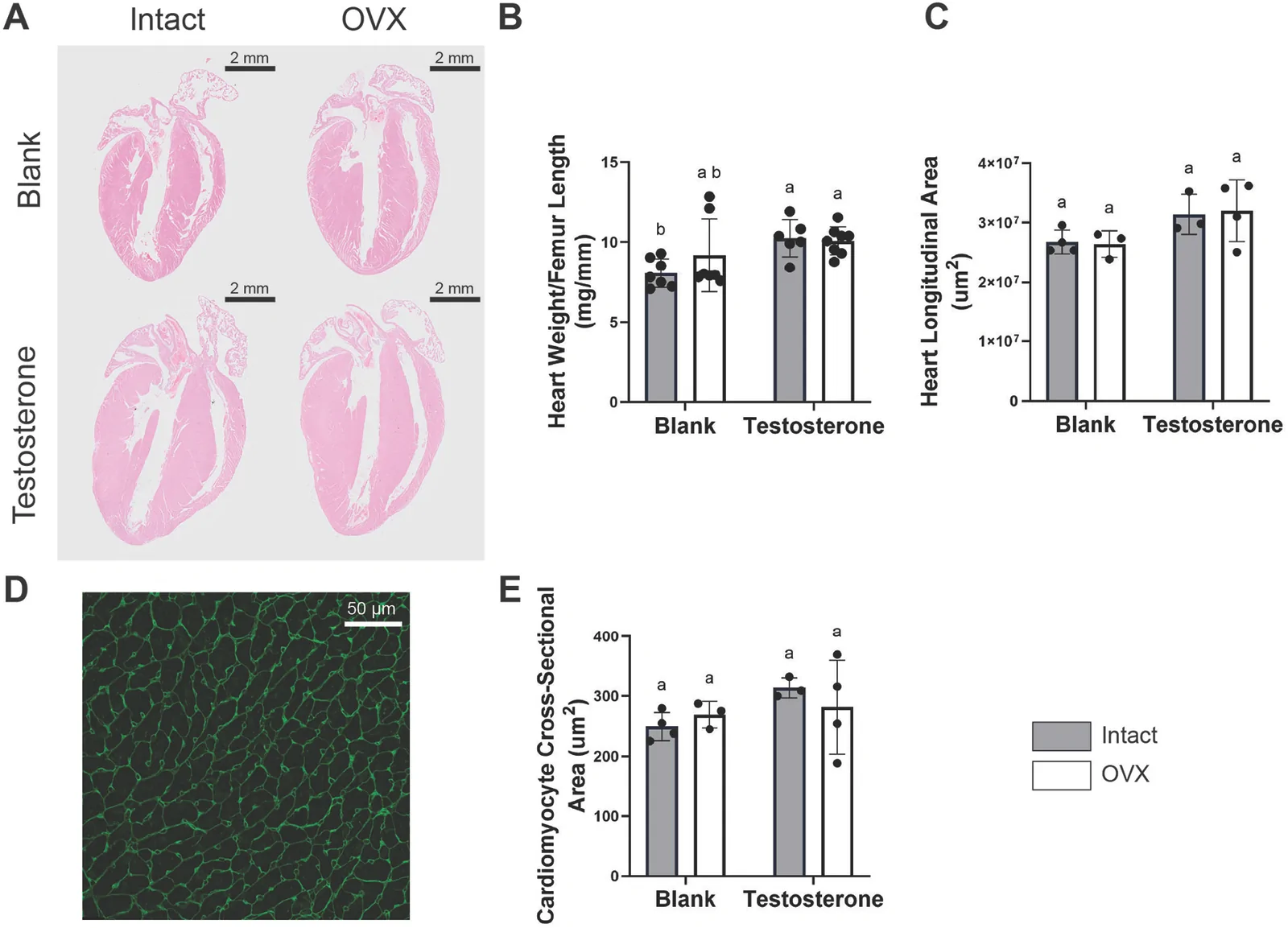
Effects of testosterone treatment on cardiac phenotypes in ovary-intact and ovariectomized mice. **(A)** Representative four-chambered longitudinal heart sections. Scale bar = 2 mm. **(B)** Average terminal heart weights (exsanguinated), normalized to femur length as in (57). **(C)** Average ventricular area of longitudinal heart sections. **(D)** Representative wheat-germ agglutinin (WGA) staining for cardiomyocytes in left ventricle. Scale bar = 50 µm. **(E)** Average cardiomyocyte cross-sectional area quantified from WGA staining. Statistical differences between groups as assessed by two-way ANOVA are represented by distinct letters (p<0.05).

### 3.5 Testosterone treatment is sufficient to prevent OVX-induced bone loss

Testosterone increased density and biomechanical strength of bone tissue regardless of gonad presence. NanoCT scan analysis revealed minimal impact of either testosterone or OVX on femoral cortical bone (**Figure 5A–B**; **Supplementary Figures 4A–F**). However, in trabecular bone both Testosterone groups had greater femoral trabecular number, less trabecular spacing, and higher bone density measures (**Figures 5C–G**; **Supplementary Figures 4G–I**). Interestingly, trabecular thickness was not increased despite the greater number of trabeculae. These data collectively suggest testosterone treatment not only prevents any OVX-associated bone loss but increases trabecular bone density even in the absence of endogenous gonads. Biomechanical testing of radii supported this conclusion, as both testosterone-treated groups showed congruent trends toward an increase in maximum load and bending stiffness in 3-point bending tests (**Figures 5H–K**; **Supplementary Figures 4J–K**).

**Figure 5:**
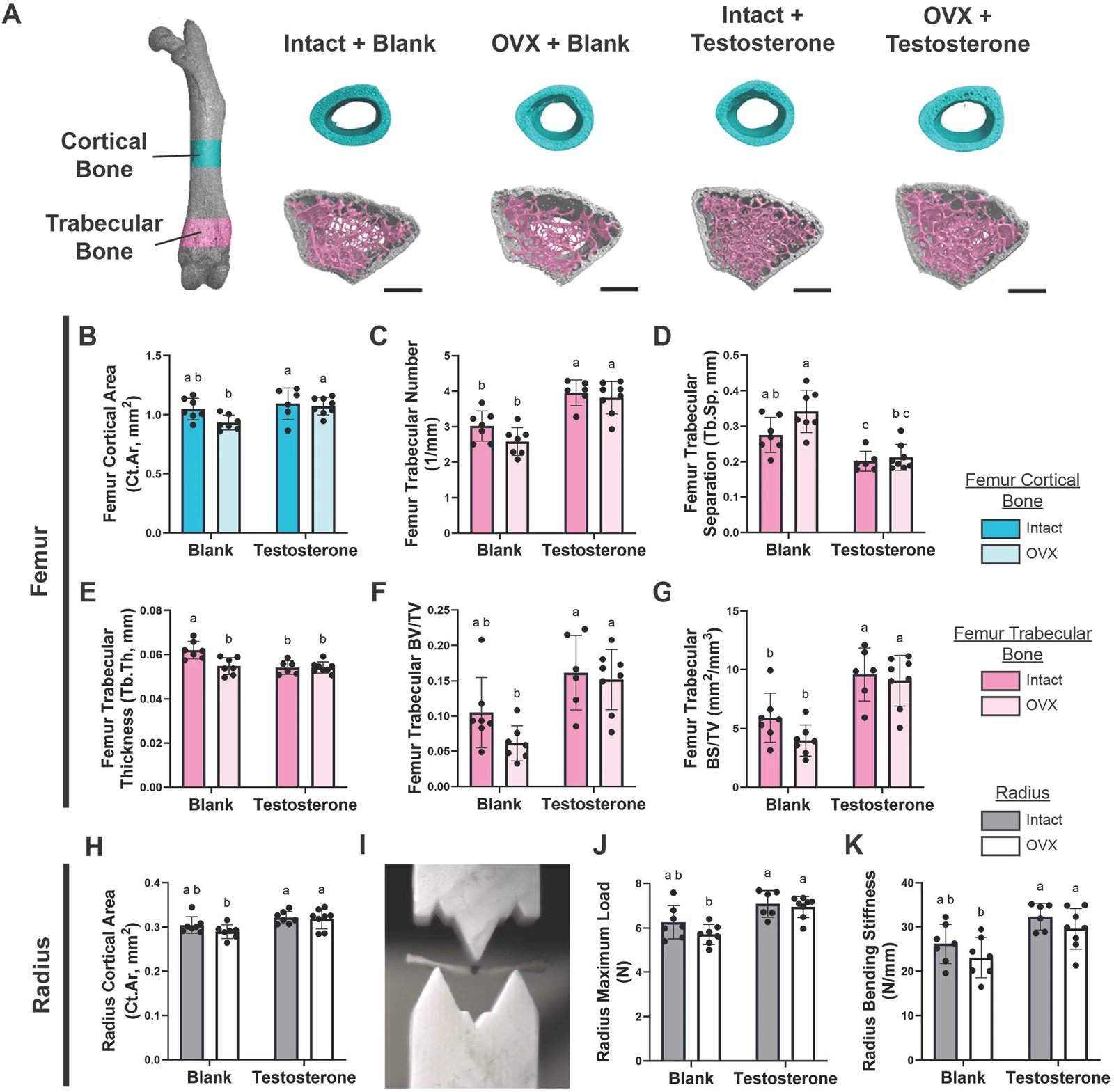
Effects of testosterone treatment on bone microstructure and biomechanics in ovary-intact and ovariectomized mice. **(A)** Representative nanoCT scans of femoral cortical and trabecular bone. **(B)** Quantification of total femoral cortical area. **(C)** Quantification of femur trabecular number, **(D)** separation, **(E)** thickness, **(F)** bone volume/trabecular volume (BV/TV), and **(G)** bone surface area/trabecular volume (BS/TV). **(H)** Quantification of radius cortical area. **(I)** Apparatus of three-point-bending test for radii. **(J)** Maximum load required for biomechanical failure of radii. **(K)** Bending stiffness of radii. Statistical differences between groups as assessed by two-way ANOVA are represented by distinct letters (p<0.05).

## 4 Discussion

Although estradiol is known to support metabolic health in females by limiting weight gain, increasing insulin sensitivity, and decreasing cholesterol levels, the role of testosterone in female metabolic homeostasis remains less well defined. Through our comparison of the impacts of testosterone treatment with and without endogenous ovaries, we show that testosterone does not influence female metabolic health outcomes through a single, uniform mechanism. For some traits, including glucose metabolism and bone health, testosterone appeared to compensate for the loss of endogenous ovarian factors. For others such as cholesterol level and adipocyte size, testosterone did not fully restore function in the absence of ovaries. Together, these findings differentiate metabolic systems that are highly sensitive to estrogen levels from those in which testosterone supplementation, and any resulting estradiol production by aromatase, may maintain homeostasis despite ovary removal.

Several metabolic endpoints, including fasting insulin levels, metabolic tissue fibrosis, and hepatic lipid accumulation, were not affected by either testosterone level or gonad presence over the 6-week treatment period of our study. These findings differ from reports that OVX promotes insulin resistance and hepatic steatosis in mice (8, 29). It is likely that differences in mouse strain and diet may account for these discrepancies; indeed, OVX mice do not always develop fatty liver on standard diet (30) and CD-1 mice are known to be resistant to hepatic steatosis even if ovariectomized (31). The absence of overt metabolic dysfunction in our OVX mice highlights the importance of genetic background and diet in determining susceptibility to reproductive hormone–associated metabolic disease.

Testosterone treatment was sufficient to regulate glucose metabolism and preserve bone density in the absence of ovaries, demonstrating that it can functionally compensate for the loss of ovarian factors for these traits. As expected, the Blank+OVX group developed impaired glucose tolerance and hyperglycemia consistent with previous studies in OVX mice (8). In contrast, Testosterone+OVX mice retained normal glucose function, demonstrating that testosterone supplementation alone may maintain glucose homeostasis. Similarly, both Testosterone groups displayed increased trabecular bone density and biomechanical strength above baseline regardless of gonad presence. These findings demonstrate that elevated testosterone is sufficient to overcome OVX-induced osteopenia. The decrease in bone density in our Blank+OVX mice did not reach statistical significance in our study, likely due to the short treatment period, but the relationship between decreased estradiol and bone loss is well-documented (32). Interestingly, testosterone increased trabecular number but not thickness, consistent with previous reports in mice (11, 33, 34) and humans (35–37). Together, these findings suggest that testosterone improves overall trabecular bone density and induces formation of more, but thinner, trabeculae. Testosterone treatment had little effect on cortical bone, supporting clinical evidence that cortical microstructure and density are not as strongly regulated by testosterone (38).

In contrast, testosterone was unable to prevent dyslipidemia in the absence of ovaries. Both Blank+OVX and Testosterone+OVX mice had elevated fasting cholesterol levels and enlarged adipocytes in inguinal fat pads. In addition, ovariectomy increased HDL cholesterol regardless of testosterone treatment, recapitulating findings from some studies of the impact of OVX (39), but not others (40, 41). As mice carry most plasma cholesterol in HDL, elevated HDL in mice is more likely to reflect overall hypercholesterolemia and is not necessarily a protective phenotype against metabolic disease as in humans (42). Similarly, the observed adipose tissue hypertrophy in OVX mice is consistent with findings after clinical oophorectomy or menopause (7, 43) and previous studies in mice (8, 30, 44). It is plausible that these effects relate to estradiol depletion, which can decrease energy expenditure, increase inflammation, and decrease lipolysis. Testosterone treatment increases energy expenditure in males, at least partially through aromatization into estradiol (45). However, our Testosterone+OVX results suggest that testosterone aromatized in non-ovarian tissues is not sufficient to maintain lipid homeostasis in females.

Our findings suggest that testosterone directly regulates heart weight and triglyceride levels through androgen signaling. Testosterone treatment alone impacted relatively few metabolic risk factors apart from increased heart weight and fasted circulating triglycerides in mice with and without ovaries. Exogenous testosterone administration is known to suppress the endogenous HPG axis and lower estradiol levels (46); however, it is unlikely that these metabolic changes were driven by reduced estradiol as Blank+OVX mice did not exhibit the same phenotypes. Likewise, though the role of aromatized testosterone cannot be ruled out without further experimentation, the fact that both testosterone-treated groups show identical deviations from untreated, intact controls suggests strongly that these changes are more likely to be androgenic effects. This premise is consistent with prior studies showing that males generally have greater heart mass (47) and higher triglyceride levels (48) compared to females and testosterone treatment can induce cardiomyocyte hypertrophy *in vitro* (49, 50).

Interestingly, Testosterone+Intact mice displayed decreased fat pad weight and median adipocyte size, despite increased circulating triglyceride levels. This pattern aligns with sexual dimorphism in body composition, where males have lower body adiposity than females; however, it does not reflect the high prevalence of obesity in PMOS females (51). As decreased adiposity was not observed in Testosterone+OVX mice, it is likely that adipose tissue changes depend on increased aromatization of testosterone into estradiol. Consistent with this, testosterone, but not DHT, reduces fat mass in female and castrated male mice, supporting a role for aromatization (45, 52). Altogether, our findings suggest that lipid metabolism is more sensitive to the level of estradiol than that of testosterone.

In interpreting these results, it is of critical importance that testosterone treatment did not appear to fully suppress estrogenic activity despite ceased estrous cyclicity. Testosterone+Intact mice showed increased uterine weight and cross-sectional area, similar to previous studies (28). The uterotrophic effect of testosterone suggests significant aromatization and estrogenic signaling, despite acyclicity. This highlights a key distinction from the hormonal environment of Blank+OVX mice, which are similarly acyclic but show uterine atrophy reflecting more severe estrogen deficiency. Interestingly, Testosterone+OVX uteri were indistinguishable from negative controls in all examined uterine outcomes, avoiding both testosterone-induced hypertrophy and OVX-induced atrophy. If the uterotrophic effect of testosterone is from aromatization into estradiol, this would indicate that peripheral tissues alone can generate bioactive levels of estradiol to sustain uterine morphology. Furthermore, the greater uterotrophic effect in Testosterone+Intact compared to Testosterone+OVX may indicate that ovaries retain significant aromatase activity despite suppression of the HPG axis by testosterone. Alternatively, uterotrophy in Testosterone+OVX mice could reflect a direct effect of testosterone in uterine tissue via AR signaling (53). Distinguishing these two pathways will be of great interest in future studies; however, either mechanism suggests that testosterone treatment and ovariectomy do not result in identical hormonal environments, despite similar repression of estrous cycling. Such differences in estrogenic activity may explain how Testosterone+Intact mice differ from OVX groups in metabolic outcomes such as adiposity and cholesterol level.

Our study faces some limitations, especially in determining the mechanisms governing our observed metabolic changes. A primary shortcoming is the inability to distinguish which effects of testosterone administration result from androgen signaling and which may be due to aromatization of testosterone. Many peripheral tissues express aromatase and this mechanism is undoubtedly at play even in ovariectomized animals. Previous studies have shown that aromatization is critical for the beneficial effects of androgen administration on rodent metabolism (54) and skeletal health (55). Future studies may employ methods such as aromatase inhibition or AR-KO models to distinguish these two important mechanisms. Further studies may also employ assays such as insulin tolerance testing, estradiol measurements, and quantification of metabolic gene expression to refine the mechanistic pathways more definitively.

Altogether, our results suggest distinct mechanisms for ovarian factors, direct effects of testosterone, and indirect effects of testosterone in regulating female metabolism and skeletal health. Clinically, these distinctions are important to women with conditions characterized by high testosterone levels, including PMOS, congenital adrenal hyperplasia, environmental exposure, or androgen-producing tumors (56). Several of these conditions are common co-morbidities with metabolic syndrome and obesity, which worsen in peri-menopause (6). This study may inform which metabolic disease factors are likely to increase in risk after estradiol depletion and which may be compensated by high testosterone. Importantly, this study may also be of interest to transmasculine patients receiving testosterone gender-affirming hormone therapy (T-GAHT), especially those considering gender-affirming oophorectomy. These results suggest that T-GAHT is sufficient to maintain normal glucose metabolism and bone density after oophorectomy, but that gonad retention may be protective with respect to lipid and adipose tissue regulation. Further research investigating mechanisms of interaction between endogenous and exogenous hormones regulating metabolic health, effects in individuals with existing disease states such as obesity, diabetes, and cardiovascular disease, and potential mitigation strategies for metabolic risk factors will be crucial to support the health of hyperandrogenic patients.

## Data Availability

The original contributions presented in the study are included in the article/**Supplementary Material**. Further inquiries can be directed to the corresponding author.

## Supplementary material

Supplementary Figure 1 Effects of testosterone treatment and ovariectomy on estrous cyclicity. Representative estrous cycling seven days before surgery and seven days after surgery for **(A)** Blank+Intact, **(B)** Blank+OVX, **(C)** Testosterone+Intact, and **(D)** Testosterone+OVX treatment groups. Red arrows indicate sustained diestrus after surgery. **(E)** Percent of mice in each group still actively cycling relative to day of surgery.

Supplementary Figure 2 Effects of testosterone treatment on other metabolic variables in ovary-intact and ovariectomized mice. **(A)** Average total body weight of mice over the treatment period. **(B)** Terminal absolute heart weight. **(C)** Terminal heart weight normalized to body weight. (D) Picro-sirius red staining for collagen in heart tissue. Whole heart image captured at 5X (scale bar = 1mm), inset at 20X (scale bar = 200 µm). **(E)** Average integrated density and **(F)** percent area of picrosirus red staining from 20X sample areas. Statistical differences between groups as assessed by two-way ANOVA are represented by asterisks or distinct letters (p<0.05). *p<0.05.

Supplementary Figure 3 Effects of testosterone treatment on liver phenotypes in ovary-intact and ovariectomized mice. **(A)** Terminal liver weights. **(B)** Representative PSR staining image of each treatment group. **(C)** Quantification of integrated density of collagen from PSR staining. **(D)** Average percent area of collagen, quantified from PSR staining. **(E)** Representative Oil Red O staining in cryopreserved liver tissue. **(F)** Average percent area of lipid droplets and **(G)** average lipid droplet size quantified from Oil Red O staining. Statistical difference as assessed by two-way ANOVA (P < 0.05) is indicated by different letters. Scale bar = 100 µm.

Supplementary Figure 4 Effects of testosterone treatment on additional bone phenotypes in ovary-intact and ovariectomized mice. **(A)** Total femur length for all groups. **(B)** Quantification of marrow area, **(C)** total area, **(D)** perimeter, **(E)** thickness, and **(F)** bone mineral density for femur cortical bone. **(G)** Quantification of connective density, structural model index, and bone mineral density for femur trabecular bone. **(J)** Quantification of total area and **(K)** marrow area for radii. Statistical difference as assessed by two-way ANOVA (P < 0.05) is indicated by different letters.
